# ITRAQ-based quantitative proteomic analysis of *japonica* rice seedling during cold stress

**DOI:** 10.1270/jsbbs.21081

**Published:** 2022-02-02

**Authors:** Dongjin Qing, Guofu Deng, Yinghua Pan, Lijun Gao, Haifu Liang, Weiyong Zhou, Weiwei Chen, Jingcheng Li, Juan Huang, Ju Gao, Chunju Lu, Hao Wu, Kaiqiang Liu, Gaoxing Dai

**Affiliations:** 1 Rice Research Institute, Guangxi Academy of Agricultural Sciences/Guangxi Key Laboratory of Rice Genetics and Breeding, Nanning, China; 2 Guangxi Academy of Agricultural Sciences/Guangxi Crop Genetic Improvement and Biotechnology Laboratory, Nanning, China

**Keywords:** iTRAQ-labeling, quantitative proteomics, cold stress, *japonica* rice

## Abstract

Low temperature is one of the important environmental factors that affect rice growth and yield. To better understand the *japonica* rice responses to cold stress, isobaric tags for a relative and absolute quantification (iTRAQ) labeling-based quantitative proteomics approach was used to detected changes in protein levels. Two-week-old seedlings of the cold tolerant rice variety Kongyu131 were treated at 8°C for 24, 48 and 72 h, then the total proteins were extracted from tissues and used for quantitative proteomics analysis. A total of 5082 proteins were detected for quantitative analysis, of which 289 proteins were significantly regulated, consisting of 169 uniquely up-regulated proteins and 125 uniquely down-regulated proteins in cold stress groups relative to the control group. Functional analysis revealed that most of the regulated proteins are involved in photosynthesis, metabolic pathway, biosynthesis of secondary metabolites and carbon metabolism. Western blot analysis showed that protein regulation was consistent with the iTRAQ data. The corresponding genes of 25 regulated proteins were used for quantitative real time PCR analysis, and the results showed that the mRNA level was not always parallel to the corresponding protein level. The importance of our study is that it provides new insights into cold stress responses in rice with respect to proteomics and provides candidate genes for cold-tolerance rice breeding.

## Introduction

Rice (*Oryza sativa*) is one of the most important food crops in the world, feeding about half of the population (Khush 2005). Climate is an important factor affecting rice yield, especially low temperatures, which affect rice growth in tropical and subtropical areas, throughout vegetative to reproductive stages (Jin *et al.* 2018). Low temperature can cause severe injury in seedlings of cold-sensitive rice cultivars in the early season, and reduce growth rate and cause pollen sterility in the late season (Cui *et al.* 2005, Nijat *et al.* 2004). Low temperature stress occurs frequently and has wide-ranging influence in the world with the increase in global climate anomalies (Cen *et al.* 2018, Pradhan *et al.* 2016). Therefore, it is important to explore genes/proteins that are regulated by low temperature in order to understanding the cold-tolerance mechanism and breeding cold-tolerant rice cultivars.

An increasing number of molecular genetic studies have elucidated how rice plants respond to low temperature stress, as well as the genes/proteins involved in the response. Low temperature is first perceived by the temperature sensor COLD1/RGA1 complex on the plasma membrane, then the complex triggers calcium influx, reactive oxygen species (ROS) production, ABA accumulation and a MAPK cascade (OsMKK6-OsMPK3) leading to active downstream transcription factors responses in the nucleus (Guo *et al.* 2018, Ma *et al.* 2015, Manishankar and Kudla 2015). Recently, the vitamin E-vitamin K1 sub-network of the COLD1 downstream pathway was found to be responsible for chilling tolerance divergence (Luo *et al.* 2021). Other components of cold tolerance have been identified recently, such as *CTB4a*, which interacts with a beta subunit of ATP synthase AtpB to mediate the ATP supply in rice plant cells to improve cold tolerance (Zhang *et al.* 2017). The standing variation of cold tolerance gene *CTB2* and *de novo* mutation of *CTB4a* facilitate cold adaptation of rice cultivation from high altitude to high latitude areas (Li *et al.* 2021); bZIP73^Jap^ in *japonica* rice cultivars interacts with bZIP71 to modulates abscisic acid (ABA) levels and reactive oxygen species (ROS) homeostasis for enhancing rice tolerance to cold climate (Liu *et al.* 2018); rice OsMADS57 interacts with OsTB1 and both directly target *OsWRKY94* and *D14* for adaptation to cold (Chen *et al.* 2018). At the rice seedling stage, the cold tolerance associated gene *qCTS-9* was identified in hybrid rice under different cold environments using QTL mapping and genome-wide expression profiling methods (Zhao *et al.* 2017). *qPSST6*, found from cold tolerance *japonica* rice variety Kongyu131with QTL mapping and Seq-BSA approach, was validated to be a functional gene relative to cold resistance (Sun *et al.* 2018). Three genes (*LOC_Os01g55350*, *LOC_Os01g55510* and *LOC_Os01g55560*) (Zhang *et al.* 2018) and 67 QTLs (Wang *et al.* 2016), which were identified by genome-wide association analysis (GWAS), were associated with cold tolerance of *indica* and *japonica* rice, respectively. In an RNA-seq comparative analysis of cold-stressed post-meiotic anther from cold-tolerant and cold-susceptible rice cultivars, a number of ethylene-related transcription factors were found to be putative regulators of cold responses (González-Schain *et al.* 2019).

Proteomics is a robust approach for the large-scale identification of proteins and has been used for profiling proteins in rice (Agrawal and Rakwal 2011). Two-dimensional gel electrophoresis (2-DE) was used to separate proteins of rice treated with cold, and cold responsive proteins were identified using mass spectrometry analysis in early proteomics studies (Hashimoto and Komatsu 2007, Huo *et al.* 2016, Imin *et al.* 2006, Ji *et al.* 2017, Yan *et al.* 2006). iTRAQ is a powerful mass spectrometry technology, which can quantify proteins’ relative expression abundance by measuring relative peak areas of MS/MS mass spectra of iTRAQ-labeled peptides (Ross *et al.* 2004). More and more cold response proteins in rice have been monitored and characterized by the iTRAQ-labeling approach. For example, differentially expressed proteins in cold stress-treated rice that are involved in photosynthesis, metabolism, transport, ATP synthesis, ROS, stress response, DNA binding and transcription, and cell growth and integrity, as well as unknown function proteins were found using iTRAQ labeling coupled with LC-MS/MS (Cen *et al.* 2018, Neilson *et al.* 2011, Wang *et al.* 2018a). Although some cold stress responsive proteins were identified by the proteomic approach in different rice cultivars, only a small number of cold-response proteins have been identified so far.

In this study, to better understand the cold tolerance mechanism of *japonica* rice, we employed the iTRAQ labeling proteomics method to investigate the proteomic response of cold stress of the *japonica* cold-resistant rice cultivar Kongyu131. Rice seedling tissues were harvested after exposure to 8°C low temperature condition for 0, 24, 48 and 72 h. iTRAQ was used for quantifying relative protein abundance, and different expression proteins were obtained at each time point by comparing to the control samples. Our results report on a large number of cold stress-regulated proteins that have not been previously identified.

## Materials and Methods

### Plant material and cold stress treatments

The *japonica* rice variety Kongyu131, which is strongly resistant to cold weather and widely planted in the northeast area of China was used in this study. Rice seedlings were grown in the growth chamber with a 16-h light (28°C)/8-h dark (25°C) condition for 2-weeks. Cold tress treatments were performed by decreasing the temperature to 8°C, and then tissues were collected and frozen in liquid nitrogen at 0 h, 24 h, 48 h and 72 h respectively, and stored at –80°C until protein extraction. For physiology experiments, 2-week-old rice seedlings were separated into groups and treated at 8°C for 4 days, and then transferred to the normal growth temperature for 3 days. For survival rate determination, the ratio of surviving plants to total plants was calculated.

### Protein extraction, digestion and iTRAQ labeling

Protein extraction was performed according to previous methods (Guo and Li 2011, Qing *et al.* 2016, Wang *et al.* 2018b) with some modifications. The frozen rice seedling tissue (0.5 g) was ground to a fine powder with mortar and pestle pre-chilled at –80°C. The tissue powder was extracted with 5 volumes (g/mL) of extraction buffer containing: 8 M urea,150 mM Tris-HCl, pH 7.6, 1.2% Triton X-100, 0.5% SDS, 5 mM ascorbic acid, 20 mM EDTA, 20 mM EGTA, 5 mM DTT, 50 mM NaF, 1 mM PMSF, 1% glycerol 2-phosphate, 1× protease inhibitor (complete EDTA free; Roche) and 2% polyvinylpolypyrrolidone. The extract was centrifuged at 110,000 × g for 2 h at 10°C to remove debris at the bottom of centrifuge tube. The total protein in the supernatant was precipitated with 3 volumes of –20°C pre-cooled acetone:methanol (12:1 v/v) for at least 2 hours. The protein pellet was collected by centrifugation at 11,000 × g for 20 min and washed two times with acetone:methanol (12:1 v/v), then re-suspended in re-suspension buffer (100 mM Tris-HCl, pH 8.0, 8 M urea). The concentration of total protein was measured by the Bradford method and the proteins sample was used for proteomics and western blot analysis.

Protein samples (200 μg) were reduced by adding 10 mM DTT and incubating at 56°C for 1 h, followed by an alkylation reaction by adding 40 mM iodoacetamide and incubating at room temperature for 30 min in the dark. To digest proteins with trypsin, urea was diluted below 2 M using 100 mM Tris-HCl (pH 8.0), then trypsin was added in the protein solution at 1:50 ratio (enzyme:protein, w/w) and incubated at 37°C overnight. Peptides were acidized by adding formic acid to end the digestion, and then centrifuged at 12,000 × g for 15 min. The supernatant was subjected to peptide purification using a Sep-Pak C18 desalting column. The peptide eluate was vacuum-dried and stored at –20°C.

For iTRAQ labeling, 100 μg of the peptide samples was used. Samples were separately labeled with different iTRAQ labeling reagents (113, 114, 115, 116) according to the manufacturer’s instructions. The labeled samples were mixed and subjected to Sep-Pak C18 desalting, then the complex mixture was fractionated using high pH reverse phase chromatography, and combined into 15 fractions. Each fraction was vacuum-dried and re-suspended in 0.1% formic acid for MS analysis.

### LC-MS/MS analysis, and protein quantification

LC-MS/MS detection was carried out on a hybrid quadrupole-TOF LC-MS/MS mass spectrometer (TripleTOF 5600, SCIEX) equipped with a nanospray source. Peptides were first loaded onto a C18 trap column (5 μm, 5 × 0.3 mm, Agilent Technologies) and then eluted into a C18 analytical column (75 μm × 150 mm, 3 μm particle size, 100 Å pore size, Eksigent). Mobile phase A (3% DMSO, 97% H_2_O, 0.1% formic acid) and mobile phase B (3% DMSO, 97% ACN, 0.1% formic acid) were used to establish a 100 min gradient comprised of: 0 min in 5% B, 65 min of 5–23% B, 20 min of 23–52% B, 1 min of 52–80% B, 80% B for 4 min, 0.1 min of 80–5% B, and a final step in 5% B for 9.9 min. A constant flow rate was set at 300 nL/min. For IDA mode analysis, each scan cycle consisted of one full-scan mass spectrum (with m/z ranging from 350 to 1500, ion accumulation time 250 ms) followed by 40 MS/MS events (m/z ranging from 100 to 1500, ion accumulation time 50 ms). The threshold for MS/MS acquisition activation was set to 120 cps for +2~+5 precusors. Former target ion exclusion was set to 18 s.

Raw data from TripleTOF 5600 was analyzed with ProteinPilot (V4.5) using the Paragon database search algorithm and the integrated false discovery rate (FDR) analysis function. Spectra files were searched against the UniProt *japonica* rice reference proteome database using the following parameters: Sample Type, iTRAQ 8plex (Peptide labeled); Cys Alkylation, Iodoacetamide; Digestion, Trypsin; Quantitate, Bias correction, and Background correction was enabled for Specific Processing; Search Effort was set to Rapid ID. Search results were filtered with unused score and false discovery rate threshold (FDR) at 1%. Decoy hits were removed, the remaining identifications were used for quantification. Proteins with a fold change of >1.2 or <0.83 and a *p*-value of <0.05 were considered to be differentially expressed (Fan *et al.* 2016).

### Bioinformatics analysis of DEPs

All DEPs were used for hierarchical cluster analysis with the Cluster 3.0 program. The DEPs were classified and grouped into different pathways according to Gene Ontology (GO) and Kyoto Encyclopedia of Genes and Genomes (KEGG). The protein-protein interaction networks were analyzed using the STRING 10 database (https://string.embl.de).

### Quantitative real time PCR and western blot analysis

To validate the MS quantification results at the transcript level, total rice seedling tissue mRNA extracted using TRIzol reagent (Invitrogen) was used for cDNA synthesis using SuperScritRIII RT First Strand Synthesis Kit (Invitrogen) according to its protocol. SYBR^®^ Premix Ex Taq^TM^ (Takara, China) was used for real-time RT-PCR, and the specific primers (Supplemental Table 1) for target genes amplification were designed using Primer Express 3.0 software. β-actin was used as an internal control gene. Three biological repeats were performed for each target gene in real-time RT-PCR.

Western blot analysis was performed according to Qing *et al.* (2016). The rabbit polyclonal antibody was raised against synthetic oligopeptides which were identified by MS, DAGDAAPPAAATTTER to make anti-A0A0N7KH91 polyclonal antibody. The peptide antibody was made commercially (GL Biochem Co., Ltd., Shanghai, China). The plant β-actin polyclonal antibody was purchased from YIFEIXUE BIO TECH. The proteins used for western blot analysis were extracted from rice seedling tissue with urea extraction buffer, separated on 15% SDS-PAGE gel, then transferred onto a polyvinylidene fluoride membrane (Millipore, USA), which was probed with the anti-A0A0N7KH91 polyclonal antibody and anti-β-actin polyclonal antibody.

## Results

### Physiological response to cold stress

**Fig. 1.**
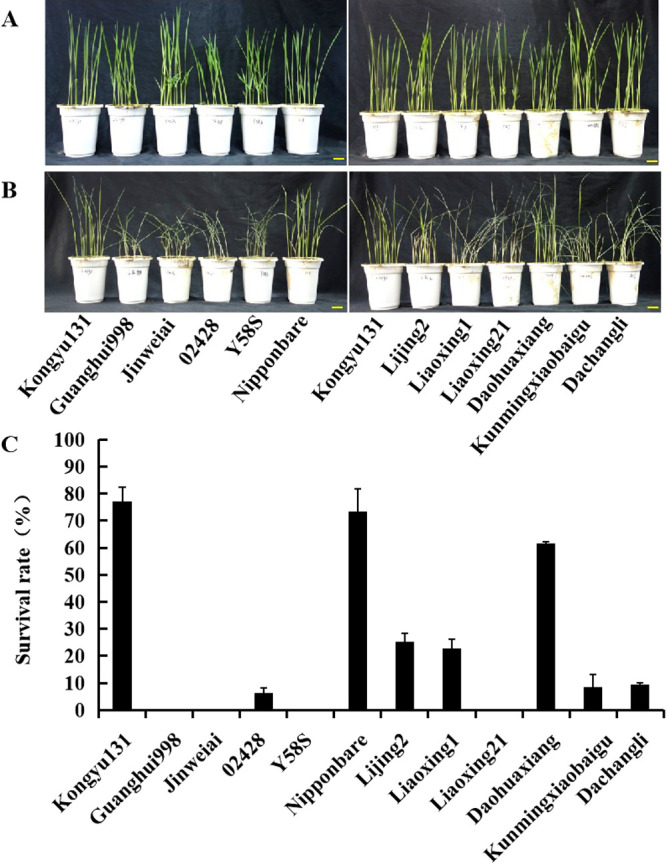
Phenotypes of cold stress treated Kongyu131 and other rice cultivars after 3 days of recovery. Rice seedling were planted in a phytotron under light and temperature controls (the day/night cycle: 12 h with 28°C and 12 h with 22°C). 2-week-old rice seedlings were treated under 8°C for 4 days and then allowed to recover for 3 days under normal temperature. (A) Phenotypes of rice cultivars before low temperature treatment. (B) Phenotypes of rice cultivars after 4 days of low temperature treatment and 3 days of recovery, bar = 2 cm. C, survival rate of rice cultivars after 3 days of recovery.

To validate contrasting stress phenotypes of Kongyu131 and 11 other cultivars in response to cold treatment, two-week-old rice seedlings were treated at 8°Cfor 4 days and then allowed to recover for 3 days. Before cold treatment, seedlings of all varieties grew normally (Fig. 1A). After the cold stress and recovery treatment, seedlings of the *indica* varieties (Guanghui998, Jinweiai, 02428, Y58S and Dachangli) and several *japonica* varieties (Liaoxing1, Liaoxing21 and Kunmingxiaobaigu) were completely wilted, whereas most seedlings of *japonica* rice varieties Kongyu131, Nipponbare and Daohuaxiang were able to survive (Fig. 1B). As seen in Fig. 1C, the survival rate of Kongyu131 after cold stress treatment was 77%, and is the highest survival rate in comparison to other varieties in this experiment.

To identify proteins that respond to cold stress in Kongyu131 at the proteomic level, two-week-old seedlings grown in soil were subjected to 0, 24, 48 and 72 h of cold stress treatments. The shoot tissues of the treated seedlings were used for quantitative proteomic analysis.

### Identification and quantitation of proteins with iTRAQ-based LC-MS/MS analysis

**Fig. 2.**
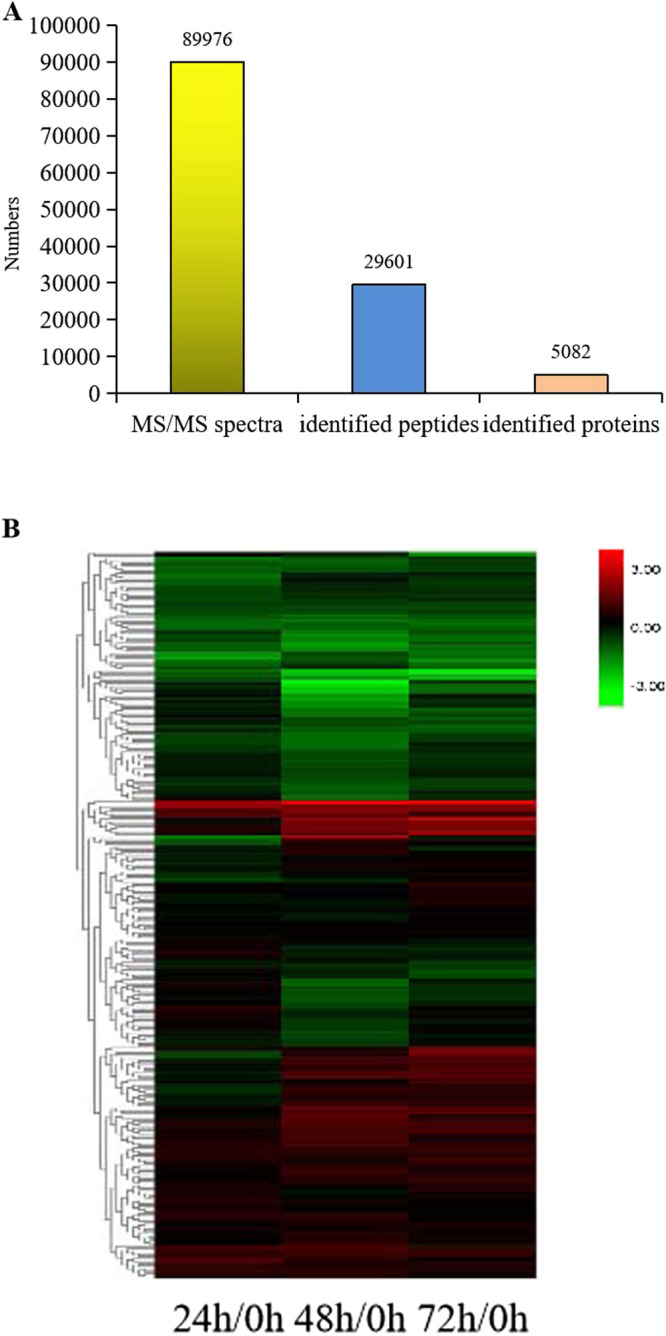
Basic information statistics of the proteome and hierarchical clustering of DEPs identified during cold stress. (A) Basic information statistics of the proteome using iTRAQ analysis. MS/MS spectra are the secondary mass spectrums, and proteins are identified by ProteinPilot (V4.5). (B) Hierarchical clustering of quantified proteins based on LC-MS/MS data.

MS raw data were analyzed with ProteinPilot (V4.5) to identify and quantify proteins. As shown in Fig. 2A, a total of 89,976 MS/MS spectra were identified by iTRAQ-based LC-MS/MS analysis in time courses of cold stress treated Kongyu131 shoot tissues. Among them, 29,601 peptides were found. At least one unique peptide was identified for each confident protein. A total of 5082 unique proteins were identified by iTRAQ labeling from the time courses of cold-stressed Kongyu131 (Supplemental Table 2).

**Fig. 3.**
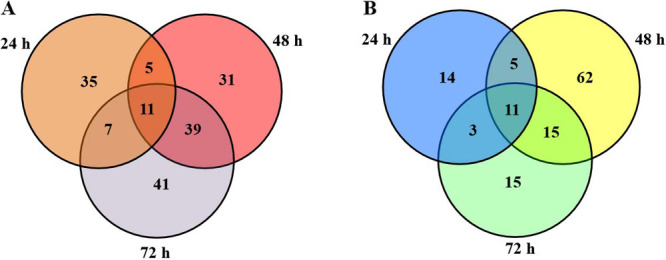
Venn diagram analysis of the differentially expressed proteins in rice at each time point of cold stress treatment. The numbers of the differentially expressed proteins identified after 24 h, 48 h and 72 h of cold treatment are shown in the different segments. (A) The up-regulated proteins. (B) The down-regulated proteins.

Through quantitative analysis with the software, 289 unique proteins were differentially expressed with changes greater than 1.2-fold or smaller than 0.83-fold, *p*-value smaller than 0.05. Regulated proteins formed two major clusters (Fig. 2B). After 24 h of cold treatment, 91 differentially expressed proteins (DEPs) (58 up- and 33 down-regulated) were found (Table 1). As the cold treatment time was increased, the number slightly increased: 179 DEPs (86 up- and 93 down-regulated) at 48 h (Table 2) and 142 DEPs (98 up- and 44 down-regulated) at 72 h (Table 3). Fig. 3 shows the Venn diagram analysis of the DEPs at different time points. Overall, there were 289 unique DEPs (169 uniquely up-regulated and 125 uniquely down-regulated) during cold stress. Among the 169 up-regulated proteins, 11 proteins were found to be significantly up-regulated at three time points (Fig. 3A), and 11 proteins of the 125 down-regulated proteins were found to be significantly down-regulated at three time points (Fig. 3B). Fifty-one proteins were found to be significantly up-regulated and 23 proteins were found to be significantly down-regulated at any two time points, respectively. Five proteins were found to be both significantly up- and down-regulated during cold stress treatments: 4 proteins up-regulated after 24 h of cold stress but down-regulated at 48 h time point, and one protein down-regulated after 48 h of cold stress but up-regulated at the 72 h time point.

### GO analysis of DEPs

**Fig. 4.**
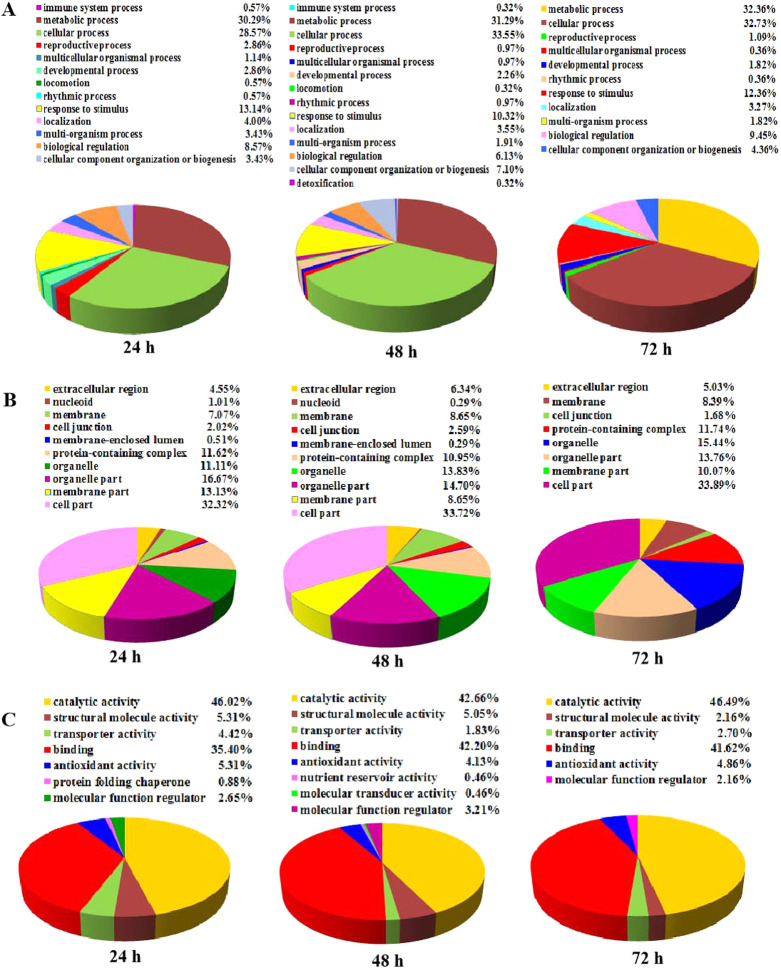
Bioinformatics analysis of DEPs in rice after 24 h, 48 h and 72 h of cold stress treatments (ratio >1.2 or <0.83). (A) Biological process. (B) Cellular component. (C) Molecular function.

To further understand the functions of DEPs, GO analysis was performed. 253 protein IDs of 289 unique DEPs were assigned functions in the GO analysis. The DEPs were significantly enriched in 13/14/11 biological processes at 24/48/72 h cold stress treatment, 10/10/8 cellular components at 24/48/72 h cold stress treatment, and 7/8/6 molecular function subgroups at 24/48/72 h cold stress treatment. The metabolic process, cellular process and response to stimulus groups were prominent in the biological process subgroup, indicating that the metabolic processes are more quickly affected under cold stress (Fig. 4A). The cell part, organelle, organelle part, membrane part and protein-containing complex groups were highly localized within the cellular component subgroup (Fig. 4B). Among the DEPs, the enriched GO terms concerning molecular function showed that DEPs were mainly associated with catalytic activity and binding, followed by the structural molecule activity, antioxidant activity and molecular function regulator (Fig. 4C).

### Pathway enrichment analysis of DEPs

**Fig. 5.**
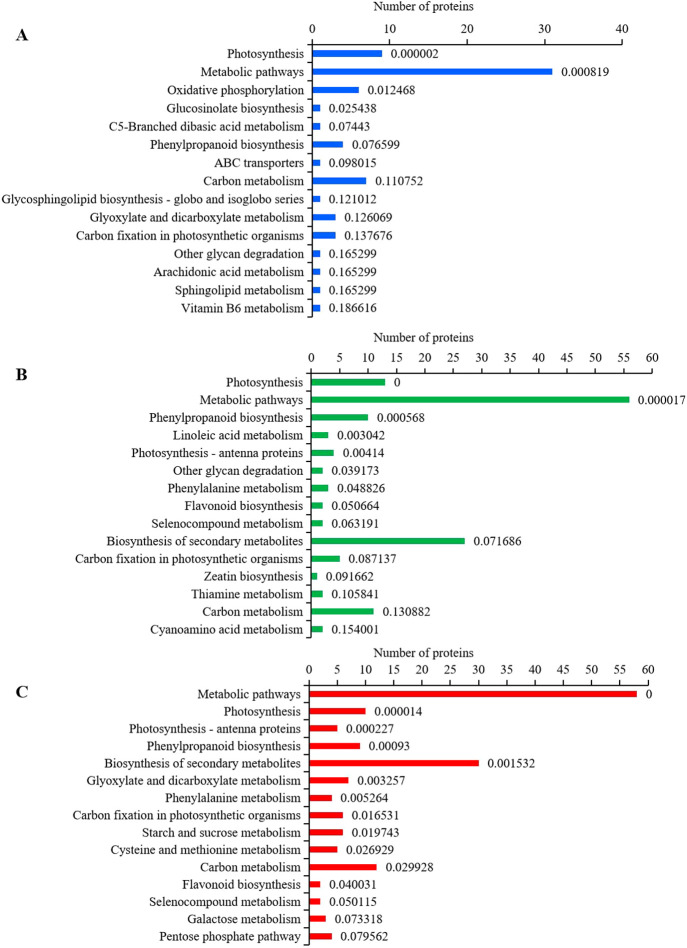
KEGG pathway analysis of DEPs in rice with cold stress treatments (ratio >1.2 or <0.83). (A) DEPs of 24 h cold treatment. (B) DEPs of 48 h cold treatment. (C) DEPs of 72 h cold treatment.

DEPs of 24 h, 48 h and 72 h cold treatment were mapped to the reference pathway in the KEGG database for functional analysis. The metabolic pathways, photosynthesis, phenylpropanoid biosynthesis, carbon metabolism and carbon fixation in photosynthetic organisms were significantly enriched in three time points of the cold stress treatment (Fig. 5). There were some different pathways enriched in different cold tress time points. For example, oxidative phosphorylation, glucosinolate biosynthesis, vitamin B6 metabolism were specifically enriched at 24 h cold stress treatment (Fig. 5A); linoleic acid metabolism, cyanoamino metabolism, thiamine metabolism and zeatin biosynthesis were specifically enriched at 48 h cold stress treatment (Fig. 5B); cysteine and methionine metabolism, galactose metabolism pentose phosphate pathway and starch/sucrose metabolism were specifically enriched at 72 h cold stress treatment (Fig. 5C). As the cold stress time increased, enriched pathways were more and more stable, with ten of the pathways affected in both the 48 h and 72 h cold stress time points (Fig. 5).

### Protein-protein interaction analysis of DEPs

**Fig. 6.**
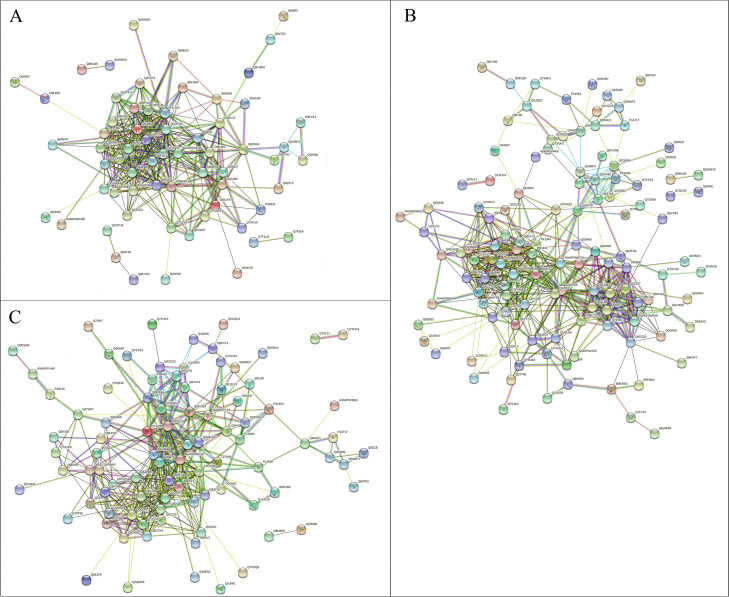
The protein-protein interaction network of cold-response proteins generated using the STRING database. Medium confidence (STRING score = 0.4) was set in the network analysis. The edges represent predicted protein-protein associations. (A) DEPs of 24 h cold stress treatment. (B) DEPs of 48 h cold stress treatment. (C) DEPs of 72 h cold stress treatment. Lake blue lines represent data from curated databases, pink lines represent data that was experimentally determined, green lines represent gene neighborhood, red lines represent gene fusions, dark blue lines represent gene co-occurrence, yellow lines represent text mining, black lines represent co-expression, sky blue lines represent protein homology.

The protein-protein interaction networks of DEPs from three time points of cold stress treatment containing biological processes, cellular components and molecular function were constructed based on the Search Tool for the Retrieval of Interacting Genes/Proteins 11.0 (STRING 11.0) database. By removing unconnected proteins, the resulting network of 24 h cold response proteins contained 64 protein nodes and 360 edges (Fig. 6A), the resulting network of 48 h cold response proteins contained 121 protein nodes and 546 edges (Fig. 6B), and the resulting network of 72 h cold response proteins contained 90 protein nodes and 402 edges (Fig. 6C). In biological processes, DEPs that function in metabolic process, cellular process, response to stimulus and biological regulation are highly up-regulated during cold stress; in terms of cellular components, DEPs that function in extracellular region, membrane, protein-containing complex and cell parts are up-regulated during cold stress; in terms of molecular function, DEPs that function in catalytic activity, binding and molecular function regulator are highly up-regulated during cold stress.

### Validation of iTRAQ data on selected candidates by Q-PCR and western blot

**Fig. 7.**
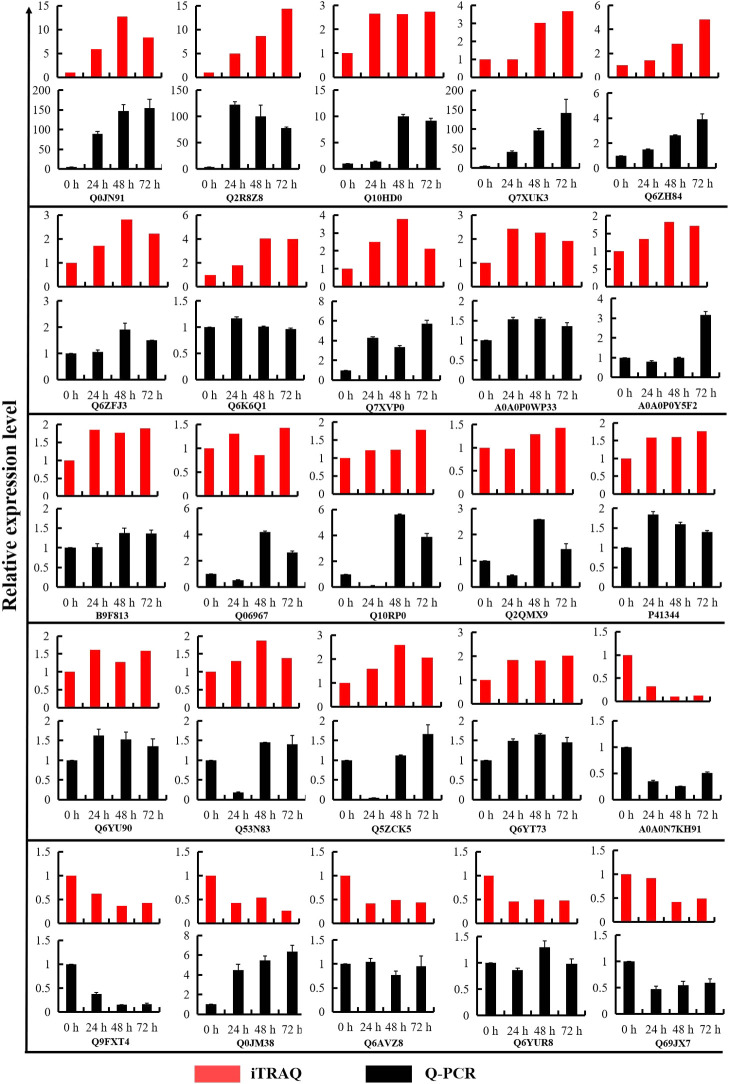
Comparative analysis of the protein and mRNA profiles of 25 representative DEPs. The X-axis represents the time points in the cold treatments. The Y-axis indicates the normalized relative protein and mRNA levels. The red and black columns represent the patterns of protein and mRNA expression in Kongyu131, respectively.

19 up-regulated DEPs and 6 down-regulated DEPs were selected for Q-PCR analysis, to validate the relationship of expression profiles between mRNA and protein level. We compared the transcription levels of 24 h, 48 h and 72 h cold stress treatments with the iTRAQ data. As shown in Fig. 7, Q-PCR data indicated that the mRNA levels of 19 up-regulated DEPs increased under cold stress, the regulation trends of four DEPs (Q7XUK3, Q6ZH84, Q6ZFJ3, A0A0P0WP33) at three time points of cold treatment were consistent with the iTRAQ quantification data. The mRNA levels of four out of six down-regulated DEPs decreased under cold stress, and the regulation trends of two DEPs (A0A0N7KH91 and Q9FXT4) at three time points of cold treatment were consistent with the iTRAQ quantification data (Fig. 7). The relative mRNA levels of some proteins were inconsistent with the iTRAQ data, maybe the expression of these genes is controlled by posttranscriptional regulation processes involving translation initiation, mRNA and protein stability (Tian *et al.* 2004).

**Fig. 8.**
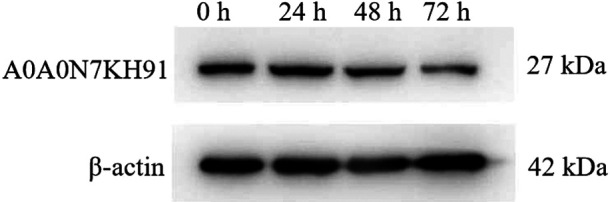
Western blot analysis of time courses of cold-treated rice cultivar Kongyu131. Equal amounts of 50 μg of total protein from different samples was used for western blot analysis using the enhanced chemiluminescence (ECL) method. The specific antibodies against A0A0N7KH91 protein (1:500) and β-actin (1:5000) were used to detect the corresponding protein expression.

To further validate the protein regulation levels of DEPs identified by the iTRAQ labeling analysis, one down-regulated protein A0A0N7KH91 was selected for confirmation by western blot analysis with a specific peptide antibody raised against the protein, and using β-actin antibody as control. Fig. 8 showed that A0A0N7KH91 protein also down-regulated during cold stress treatment from 24 h to 72 h. This result further confirmed the iTRAQ labeling analysis data.

## Discussion

In this work, we performed quantitative proteomic analysis of *japonica* rice seedlings subjected to time course cold stress treatments to obtain the dynamic proteins expression patterns responsive to low temperature. Using iTRAQ labeling coupled with LC-MS/MS analysis, 5802 proteins were identified and were used for quantification from the rice tissues. As a result, we found 91/179/142 cold responsive proteins at 24/48/72 h cold stress treatments with the fold change >1.2 or <0.83 with a *p*-value <0.05 for the differentially regulated proteins (Tables 1–3), and the number of cold responsive proteins increased when the treatment time increased. This result is consistent with a previous quantitative proteomic analysis of *indica* rice that also used a time course of cold stress treatment (Wang *et al.* 2018a).

Increasing proteomics studies on rice cold stress treatment are being used to explore cold response proteins for understanding plant cold-tolerance mechanism. Some proteins that were identified previously to be cold response proteins have been further confirmed in our study using the quantitative proteomic method. These proteins mainly include: sucrose synthase (Cui *et al.* 2005, Maraña *et al.* 1990), phenylalanine ammonia-lyse (Cui *et al.* 2005, Leyva *et al.* 1995, Sanchez-Ballesta *et al.* 2000), GSTs (Binh and Oono 1992, Cui *et al.* 2005, Marrs 1996, Roxas *et al.* 1997), 14-3-3 like protein GF14-F/Drought induced protein 3/Peroxidase/Phosphoserine aminotransferase (Wang *et al.* 2018a), ATP synthase (Cen *et al.* 2018, Cui *et al.* 2005, Ji *et al.* 2017, Wang *et al.* 2018a, Yan *et al.* 2006), cold shock domain protein (Chaikam and Karlson 2008), drought-induced S-like ribonuclease (Neilson *et al.* 2011), DUF26-like protein/Photosystem related proteins/Non-specific lipid-transfer protein (Cen *et al.* 2018, Wang *et al.* 2018a), and malate dehydrogenase (Lee *et al.* 2009, Yan *et al.* 2006).

Based on an analysis of the physiological functions of cold responsive proteins in previous studies, some relative to cold genes identified in the present work may serve to resist cold stress. 14-3-3 proteins can regulate target proteins involved in responses to biotic and abiotic stress through protein interactions (Chen *et al.* 2006, Cooper *et al.* 2003), and it also may enhance tolerance to abiotic stress by ion channels regulation and hormone signaling pathways participation (Zhao *et al.* 2021). 14-3-3-like protein GF14c can target to plasma, thylakoid and vacuolar membranes and associated ATPase synthase complexes involved in stress responses (Baunsgaard *et al.* 1998, Cooper 2001, Sehnke *et al.* 2000). 14-3-3-like protein GF14-F (Q06967) was shown to be up-regulated during cold stress in this study, and probably functions in cold resistant in the cold tolerance rice cultivar Kongyu131. Calcium-transporting ATPase genes differentially expressed under cold, salt and drought stresses are involved in abiotic stress signaling (Singh *et al.* 2014), the calcium-transporting ATPase 10 protein (Q2QMX9) up-regulated after 72 h cold stress treatment might trigger a stress signaling pathway. Chaperone protein ClpD1 is involved in heat and osmotic stress response, and up-regulation of this protein is correlated with increased drought tolerance in rice (Wu *et al.* 2016). Thus, the up-regulation of chaperone protein (Q6H795) both at 48 h and 72 h of cold stress treatment might play a role in resisting cold stress in this experiment. 6-phosphogluconate dehydrogenase activity increased in rice seedlings during various abiotic stresses treatments, and might function as a regulator to control the efficiency of the pathway under abiotic stresses (Hou *et al.* 2007). Thus, 6-phosphogluconate dehydrogenase (Q2R480) up-regulated maybe regulate the pathway efficiency under cold stress in this experiment. Cold shock domain proteins can have inducible expression under cold stress conditions for cold acclimation, as seen in *Arabidopsis* and winter wheat (Fusaro *et al.* 2007, Karlson and Imai 2003, Karlson *et al.* 2002). Cold shock domain proteins do not accumulated during low temperature stress treatment in rice (Chaikam and Karlson 2008), and in the present study, the cold shock domain protein 1 (Q6YUR8) down-regulated both at 48 h and 72 h cold stress treatments (Tables 1–3). Thus, the protein might have different regulation in different rice varieties, which have different resistant levels to cold stress. These correlative data support the notion that the protein might involved in the cold acclimation response.

Interesting, in the 289 DEPs, only 11 proteins continued to be up-regulated and 11 proteins continued to be down-regulated from 24 h to 72 h of cold stress treatment. These continuously regulated proteins during cold stress could play and important role for rice cold resistance. Three proteins (cell division cycle protein, GLO1 and GLO5) have been reported relative to cold stress in previous studies. Cell division cycle protein 48 (CDC48) is associated with leaf senescence and plant survival in rice (Huang *et al.* 2016), and a single base substitution in yeast CDC48 can change yeast sensitivity to cold stress and cause cell death (Madeo *et al.* 1997). A homolog of AtCDC48, AtOM66 which is located on the outer mitochondrial membrane in *Arabidopsis* plays a role in regulating cell death in response to biotic and abiotic stresses (Zhang *et al.* 2014). In the present study, CDC48 protein (Q10RP0) up-regulation may play role in enhancing survival of Kongyu131 by determining the progression of cell death during cold stress treatment. In a previous study, Glycolate oxidase (GLO) potentially interacted with catalase (CAT) to regulate H_2_O_2_ levels in rice under environmental stress or stimuli (Zhang *et al.* 2016). In the present study, GLO1 (Q10CE4) and GLO5 (Q6YT73) proteins up-regulation may affect H_2_O_2_ levels in Kongyu131 to enhance cold resistance. Although other DEPs identified in the work were not reported to function in cold stress response, these up- or down-regulated proteins under cold stress can now be annotated as “cold-regulated proteins”. The physiological functions of these DEPs will need to by fully characterized in future studies to enhance our understanding of cold stress responses in plants at the molecular level.

In conclusion, this study is the first to adopt iTRAQ-based quantitative proteomics approach to identify cold response proteins in cold-tolerance *japonica* rice cultivar Kongyu131. A total of 289 DEPs were identified in time courses cold stress treatment. Partial DEPs related to cold genes were also identified in this study, 14-3-3 proteins, cold shock domain protein, calcium-transporting ATPase, 6-phosphogluconate dehydrogenase, CDC48 protein and GLO1/5. Some unknown function DEPs were first be identified in this study, specially continue up-regulated proteins (Q0JN91, Q6YU90, B9F813, A0A0P0Y5F2) and continue down-regulated proteins (A0A0N7KH91, Q0D5S1) from 24 h to 72 h cold stress treatment may play an important role during cold tolerance of Kongyu131. Some DEPs were not identified in previous studies can provide candidate genes for biological function study to better understand the cold-tolerance mechanism of rice responses to cold stress. Uncover the function of these genes may provide candidate genes for cold-tolerance rice molecular breeding in future.

## Author Contribution Statement

D.Q., G.D. (Gaoxing Dai) and G.D. (Guofu Deng) contributed to experimental design. D.Q. and K.L. contributed to the protein extraction, peptide preparation, iTRAQ labeling experiments and analyzed the quantitative proteomics data; Y.P. and L.G. contributed to Q-PCR experiment; Y.P., H.L., W.C., C.L. and J.H. contributed to the rice cold stress treatment experiment; W.Z., J.G., J.L. and H.W. contributed to the western blot experiment; D.Q., G.D. (Guofu Deng) and G.D. (Gaoxing Dai) wrote the manuscript; G.D. (Gaoxing Dai) contributed to modification of the manuscript.

## Supplementary Material

Supplemental Table 1

Supplemental Table 2

## Figures and Tables

**Table 1.** List of differentially expressed proteins after cold stress treatment for 24 h

Uniprot_ID	Gene ID	Putative function	Peptides (95%)*^a^*	Cov (95%)*^b^*	Fold change
Q06967	LOC_Os03g50290	14-3-3-like protein GF14-F	28	74.62	1.31
Q6ER94	LOC_Os02g33450	2-Cys peroxiredoxin BAS1, chloroplastic	17	49.81	1.56
Q10N97	LOC_Os03g16960	33 kDa secretory protein, putative, expressed	6	35.85	0.41
O65037	LOC_Os01g69950	50S ribosomal protein L27, chloroplastic	4	22.05	1.57
Q7XR19	LOC_Os04g39700.1	60S ribosomal protein L6	13	54.05	1.54
P0DKK7	LOC_Os09g32976	60S ribosomal protein L7a-2	14	42.25	1.38
Q5N6W3	LOC_Os01g67720.1	ABC1-like	8	22.59	0.42
Q53JF7	LOC_Os11g06720	Abscisic stress-ripening protein 5	4	16.67	0.41
Q9FXT4	LOC_Os10g35110	Alpha-galactosidase	11	24.66	0.62
Q10A56	LOC_Os10g05069	Alpha-mannosidase	20	22.71	0.62
Q2QX58	LOC_Os12g07110	AMP-binding enzyme family protein, expressed	19	27.79	0.60
Q5WAB3	LOC_Os06g07090.1	AP-1 complex subunit gamma	8	11.03	0.66
Q84PA4	LOC_Os03g17070	ATP synthase B chain, chloroplast, putative, expressed	21	45.97	1.66
Q7G3Y4	LOC_Os10g17280	ATP synthase gamma chain, mitochondrial, putative, expressed	12	43.21	1.58
Q7XXS0	LOC_Os08g37320.1	ATP synthase subunit d, mitochondrial	19	67.46	1.42
Q7F354	LOC_Os01g51570.1	Beta-1,3-glucanase	7	30.18	0.46
Q10RP0	LOC_Os03g05730	Cell division cycle protein 48, putative, expressed	39	53.40	1.21
Q8RU06	LOC_Os10g22520	Cellulase containing protein, expressed	10	20.39	0.55
Q53N83	LOC_Os11g13890	Chlorophyll a-b binding protein, chloroplastic	22	51.59	1.31
Q84PB4	LOC_Os08g44680.1	Chloroplast photosystem I reaction center subunit II-like protein	25	70.44	1.77
Q69S39	LOC_Os07g37030	Cytochrome b6-f complex iron-sulfur subunit, chloroplastic	12	46.22	1.79
Q5WMX0	LOC_Os05g15770.1	DIP3	16	36.36	1.75
Q6L4S0	LOC_Os05g51480	DNA damage-binding protein 1	14	13.58	0.64
P29545	LOC_Os07g46750	Elongation factor 1-beta	10	57.14	1.41
Q851Y8	LOC_Os03g63410	Elongation factor Tu	20	48.12	1.64
Q2QN11	LOC_Os12g39360	Eukaryotic aspartyl protease family protein, expressed	10	25.34	0.44
Q2R8Z8	LOC_Os11g10470	Expressed protein	1	13.86	5.01
Q0J8M2	LOC_Os08g01380	Ferredoxin-1, chloroplastic	4	40.29	0.30
P41344	LOC_Os06g01850	Ferredoxin—NADP reductase, leaf isozyme 1, chloroplastic	30	56.08	1.58
Q6ZFJ3	LOC_Os02g01340	Ferredoxin—NADP reductase, leaf isozyme 2, chloroplastic	29	54.37	1.71
Q40677	LOC_Os11g07020	Fructose-bisphosphate aldolase, chloroplastic	47	57.73	1.50
Q10CU9	LOC_Os03g53800	Glycosyl hydrolase family 3 N terminal domain containing protein, expressed	17	28.32	0.60
Q10CU4	LOC_Os03g53860	Glycosyl hydrolase family 3 N terminal domain containing protein, expressed	19	23.96	0.52
Q84TA3	LOC_Os03g60460	Leucine aminopeptidase	15	25.20	0.64
Q8GS76	LOC_Os07g44780.1	Lipase-like protein	1	3.84	0.65
Q0D5P8	LOC_Os07g36080	Oxygen-evolving enhancer protein 3, chloroplastic	22	58.06	2.33
Q7F1U0	LOC_Os07g48020.1	Peroxidase	17	51.74	0.50
Q9FYP0	LOC_Os01g19020.1	Peroxidase	10	31.99	0.47
Q6AVZ8	LOC_Os05g04380.1	Peroxidase	16	49.12	0.42
Q0DCP0	LOC_Os06g20150.1	Peroxidase (Fragment)	14	39.53	0.53
Q10CE4	LOC_Os03g57220	Peroxisomal (S)-2-hydroxy-acid oxidase GLO1	41	69.65	1.56
Q6YT73	LOC_Os07g05820	Peroxisomal (S)-2-hydroxy-acid oxidase GLO5	38	65.85	1.84
A0A0P0WP33	LOC_Os05g41640.1	Phosphoglycerate kinase	41	68.18	2.42
Q8LMR0	LOC_Os03g06200	Phosphoserine aminotransferase	10	26.76	1.72
Q8GT95	LOC_Os07g38130	Polygalacturonase inhibitor 1	8	32.83	0.54
Q6AT26	LOC_Os05g08370	Probable cellulose synthase A catalytic subunit 1 [UDP-forming]	5	5.30	1.39
Q53LQ0	LOC_Os11g09280	Protein disulfide isomerase-like 1-1	26	41.80	1.46
Q60E59	LOC_Os05g32220.1	Ribosomal protein	17	39.83	1.45
A3BLC3	LOC_Os07g38300	Ribosome-recycling factor, chloroplastic	23	57.14	1.49
Q10M12	LOC_Os03g21040	Ricin B-like lectin R40C1	15	53.45	1.32
Q0JPA6	LOC_Os01g13210	Salt stress root protein RS1	17	66.18	1.79
Q9S827	LOC_Os08g02640	Succinate dehydrogenase [ubiquinone] iron-sulfur subunit 1, mitochondrial	6	28.47	2.65
Q0D840	LOC_Os07g08840	Thioredoxin H1	9	54.92	1.67
Q6ATY4	LOC_Os05g33280	UPF0603 protein Os05g0401100, chloroplastic	15	36.12	1.45
A0A0N7KH91	LOC_Os03g22490.1	Os03g0345700 protein (Fragment)	1	6.67	0.32
Q0DJB9	LOC_Os05g23740.1	Os05g0303000 protein (Fragment)	33	46.19	1.47
Q0DEV8	LOC_Os06g04150.1	Os06g0132400 protein (Fragment)	7	43.12	1.64
A0A0P0X3W0	LOC_Os07g13280.1	Os07g0237100 protein (Fragment)	5	13.53	1.82
Q0JAK9	LOC_Os04g50204.1	OSJNBa0009P12.17 protein	11	13.91	1.71
Q7XPV4	LOC_Os04g58710.1	OSJNBa0088H09.2 protein	15	38.22	0.46
Q0JN91	LOC_Os01g21250.1	Os01g0314800 protein	1	8.60	5.92
Q943W1	LOC_Os01g31690.1	Os01g0501800 protein	36	66.07	2.07
Q5VPC8	LOC_Os01g46510.1	Os01g0653800 protein	8	8.50	0.62
Q8W0E7	LOC_Os01g52230.1	Os01g0720400 protein	1	3.65	5.20
Q5JKX9	LOC_Os01g72049.1	Os01g0949060 protein	3	8.61	0.62
Q6YU90	LOC_Os02g01150.1	Os02g0101500 protein	42	81.61	1.61
Q6Z702	LOC_Os02g03260.1	Os02g0125100 protein	19	46.30	1.47
A0A0P0VH79	LOC_Os02g15750.1	Os02g0257300 protein	10	17.20	1.92
Q6K4S7	LOC_Os02g18410.1	Os02g0285300 protein	12	41.81	1.67
Q6Z875	LOC_Os02g21970.1	Os02g0325100 protein	12	29.12	1.61
A0A0P0VJP6	LOC_Os02g31160.1	Os02g0516800 protein	2	4.04	0.64
Q69S79	LOC_Os02g36570.1	Os02g0575500 protein	3	4.72	0.56
Q0E032	LOC_Os02g37060.1	Os02g0581100 protein	3	10.92	0.51
Q6ZGJ8	LOC_Os02g52940.1	Os02g0768600 protein	22	64.69	1.45
Q6KA00	LOC_Os02g57670.1	Os02g0822600 protein	15	55.08	1.29
B9F813	LOC_Os04g16680.1	Os04g0234600 protein	38	57.91	1.85
Q0JD53	LOC_Os04g35140.1	Os04g0430700 protein	3	4.89	0.29
Q0JAF4	LOC_Os04g51300.1	Os04g0602100 protein	19	43.63	1.98
A0A0P0WG85	LOC_Os04g56740.1	Os04g0663100 protein	15	16.69	1.63
A0A0P0WKD6	LOC_Os05g22614.1	Os05g0291700 protein	41	69.29	1.74
Q60EA3	LOC_Os05g42350.1	Os05g0503300 protein	20	29.49	1.38
Q658I3	LOC_Os06g03770.1	Os06g0128300 protein	5	6.41	0.52
Q67W57	LOC_Os06g43850.1	Os06g0646500 protein	17	56.00	1.82
A0A0P0X7J0	LOC_Os07g35520.1	Os07g0539400 protein	6	11.32	0.48
Q0D5S1	LOC_Os07g35560.1	Os07g0539900 protein	14	28.24	0.46
Q6ZG03	LOC_Os08g17390.1	Os08g0276100 protein	12	36.03	1.42
Q6YW78	LOC_Os08g29370.1	Os08g0382400 protein	26	47.42	1.77
Q8LNF2	LOC_Os10g35810	Os10g0502000 protein	12	45.34	1.37
A0A0P0Y5F2	LOC_Os11g46000.1	Os11g0687200 protein	8	12.37	1.34
Q2QWN3	LOC_Os12g08770	Os12g0189400 protein	13	53.02	0.67
Q2QSR7	LOC_Os12g23180	Os12g0420200 protein	32	55.05	1.29

*^a^* Peptides (95%) indicate the identified peptides having at least 95% confidence. *^b^* Cov (95%) indicate percentage of matching amino acids from identified peptides having at least 95% confidence.

**Table 2.** List of differentially expressed proteins after cold stress treatment for 48 h

Uniprot_ID	Putative function	Peptides (95%)*^a^*	Cov (95%)*^b^*	Fold change
Q8S6N5	Acetyl-CoA carboxylase 1	32	15.26	1.47
Q0J709	ACT domain-containing protein DS12, chloroplastic	11	44.17	2.03
Q10P83	Acyl-CoA-binding domain-containing protein 5	1	4.39	1.63
Q7F270	ADP-ribosylation factor 1 OS = *Oryza sativa* subsp. *japonica* OX = 39947 GN = OJ1118_B03.103 PE = 2 SV = 1	7	27.07	2.19
Q7XYS3	Allene oxide synthase 2	12	29.08	1.72
Q9FXT4	Alpha-galactosidase	11	24.66	0.37
Q10A56	Alpha-mannosidase	20	22.71	0.40
Q2R3E0	Alpha-mannosidase	32	33.92	0.34
Q2QX58	AMP-binding enzyme family protein, expressed	19	27.79	0.61
Q5WAB3	AP-1 complex subunit gamma	8	11.03	0.75
P12085	ATP synthase subunit beta, chloroplastic	57	75.10	2.47
Q655S1	ATP-dependent zinc metalloprotease FTSH 2, chloroplastic	35	47.34	2.23
Q2QZU5	Auxin-repressed protein-like protein ARP1, putative, expressed	3	45.60	0.19
Q75I93	Beta-glucosidase 7	12	24.80	0.48
Q0JR25	Bowman-Birk type bran trypsin inhibitor	8	33.86	0.19
A5HEI2	Bowman-Birk type proteinase inhibitor A	15	43.82	0.19
Q5VS79	Calmodulin-binding protein-like	17	33.89	0.59
B9EXM2	Carbamoyl-phosphate synthase large chain, chloroplastic	29	26.37	0.60
Q75HY2	Carboxypeptidase	11	31.45	1.46
Q10RP0	Cell division cycle protein 48, putative, expressed	39	53.40	1.24
Q8RU06	Cellulase containing protein, expressed	10	20.39	0.49
Q84T92	Chalcone—flavonone isomerase	11	53.65	2.61
Q6H795	Chaperone protein ClpD1, chloroplastic	11	14.29	1.96
Q6ZF30	Chlorophyll a-b binding protein, chloroplastic	10	51.13	4.53
Q7XV11	Chlorophyll a-b binding protein, chloroplastic	15	48.41	2.29
Q53N83	Chlorophyll a-b binding protein, chloroplastic	22	51.59	1.87
Q6H748	Chlorophyll a-b binding protein, chloroplastic	16	31.97	1.87
Q7XC09	Chloroplast chaperonin 10, putative, expressed	8	54.29	0.60
Q84PB4	Chloroplast photosystem I reaction center subunit II-like protein	25	70.44	2.11
Q5W6F1	Cinnamate-4-hydroxylase	8	15.00	2.11
Q6YUR8	Cold shock domain protein 1	11	68.88	0.50
Q7XCS3	Cys/Met metabolism PLP-dependent enzyme family protein, expressed	5	11.42	2.61
P12123	Cytochrome b6	5	25.12	3.66
Q6ZAA5	D-3-phosphoglycerate dehydrogenase	10	17.23	1.80
Q5WMX0	DIP3	16	36.36	0.63
Q306J3	Dirigent protein	16	58.50	2.88
Q69JX7	Drought-induced S-like ribonuclease	3	13.10	0.42
Q8S3P3	DUF26-like protein	10	44.96	0.29
O64937	Elongation factor 1-alpha	29	48.77	0.23
Q5Z627	Elongation factor 1-gamma 3	21	43.51	0.50
Q6ZI53	Elongation factor Tu	41	61.88	0.43
Q2QN11	Eukaryotic aspartyl protease family protein, expressed	10	25.34	0.37
Q8S7Q0	Eukaryotic translation initiation factor 3 subunit B	17	24.76	0.63
Q0DHB7	Expansin-A4	1	3.66	0.27
Q2R8Z8	Expressed protein	1	13.86	8.63
Q10T66	Expressed protein	9	49.75	0.68
Q7G649	Expressed protein	15	61.82	0.36
P41344	Ferredoxin—NADP reductase, leaf isozyme 1, chloroplastic	30	56.08	1.60
Q6ZFJ3	Ferredoxin—NADP reductase, leaf isozyme 2, chloroplastic	29	54.37	2.81
Q6ZD89	Flavone 3ʹ-O-methyltransferase 1	20	60.60	1.82
Q5N725	Fructose-bisphosphate aldolase 3, cytoplasmic	28	72.07	0.74
Q5VQG4	Galactinol—sucrose galactosyltransferase	6	8.94	2.01
Q6Z2T6	Geranylgeranyl diphosphate reductase, chloroplastic	26	47.08	0.67
Q6ZBZ2	Germin-like protein 8-14	4	15.96	0.42
Q10CU9	Glycosyl hydrolase family 3 N terminal domain containing protein, expressed	17	28.32	0.58
Q10CU4	Glycosyl hydrolase family 3 N terminal domain containing protein, expressed	19	23.96	0.51
Q7XU02	Glycosyltransferase	3	7.34	2.17
Q5VME5	Glycosyltransferase	11	31.19	2.00
Q2R1S1	Harpin binding protein 1, putative, expressed	9	31.11	1.63
Q7XUC9	Histone H4	12	58.25	1.89
O64437	Inositol-3-phosphate synthase 1	4	8.43	2.27
Q84TA3	Leucine aminopeptidase	15	25.20	0.64
Q03200	Light-regulated protein, chloroplastic	3	36.72	0.18
P29250	Linoleate 9S-lipoxygenase 2	17	23.79	1.74
Q8GS76	Lipase-like protein	1	3.84	0.77
Q2QNN5	Lipoxygenase	23	28.74	7.80
Q7XUG1	Malate synthase	14	29.81	0.47
Q75M18	Methionine S-methyltransferase	11	12.18	1.64
Q2QM23	Methyl-CpG binding domain containing protein, expressed	13	56.44	0.39
Q7XUK3	NADPH oxidoreductase	10	36.23	3.02
Q2QYL3	Non-specific lipid-transfer protein 3	7	48.76	0.16
Q75M32	Peptidyl-prolyl cis-trans isomerase	23	60.40	0.50
Q6ZH98	Peptidyl-prolyl cis-trans isomerase	16	55.23	0.50
Q5Z9H9	Peptidyl-prolyl cis-trans isomerase	17	55.91	0.39
Q7F1F2	Peptidylprolyl isomerase	22	36.38	0.72
Q7XSV2	Peroxidase	18	51.16	0.67
Q0IMX5	Peroxidase	9	26.80	0.61
Q7XSU8	Peroxidase	12	48.53	0.55
Q6AVZ8	Peroxidase	16	49.12	0.49
Q7XSU7	Peroxidase	14	35.51	0.47
Q5Z7J2	Peroxidase	13	30.86	0.30
A0A0P0XR31	Peroxidase (Fragment)	12	32.14	0.55
Q0DCP0	Peroxidase (Fragment)	14	39.53	0.40
Q6YT73	Peroxisomal (S)-2-hydroxy-acid oxidase GLO5	38	65.85	1.82
Q6K6Q1	Phenylalanine ammonia-lyase	23	30.08	4.02
P14717	Phenylalanine ammonia-lyase	44	55.35	2.88
Q75W16	Phospho-2-dehydro-3-deoxyheptonate aldolase 2, chloroplastic	14	25.60	2.42
A0A0P0WP33	Phosphoglycerate kinase	41	68.18	2.27
Q6Z8F4	Phosphoribulokinase	27	57.32	1.53
Q8LMR0	Phosphoserine aminotransferase	10	26.76	1.77
P0C355	Photosystem I P700 chlorophyll a apoprotein A1	19	16.80	1.51
P0C364	Photosystem II CP47 reaction center protein	24	31.89	2.54
P0C434	Photosystem II protein D1	18	31.73	2.99
Q8L6I2	Plasma membrane ATPase	20	21.32	1.58
Q8GT95	Polygalacturonase inhibitor 1	8	32.83	0.32
Q6ZC69	Probable adenylate kinase 2, chloroplastic	9	33.45	1.80
Q5ZCK5	Probable calcium-binding protein CML16	5	31.49	2.58
Q75L11	Probable histone H2A.6	6	30.13	4.37
Q53RB0	Probable linoleate 9S-lipoxygenase 4	14	16.42	1.84
Q6K439	Probable plastid-lipid-associated protein 2, chloroplastic	17	42.01	1.66
Q5VMJ3	Profilin LP04	6	58.78	0.70
Q7XKF3	Protochlorophyllide reductase A, chloroplastic	17	48.06	0.31
Q6YZX6	Putative aconitate hydratase, cytoplasmic	39	41.98	0.68
A0A0P0WK98	Ribosomal protein L15 (Fragment)	9	33.82	0.59
A3BLC3	Ribosome-recycling factor, chloroplastic	23	57.14	0.59
Q10M12	Ricin B-like lectin R40C1	15	53.45	0.55
P31924	Sucrose synthase 1	63	54.90	0.59
Q7XXS4	Thiamine thiazole synthase, chloroplastic	23	42.82	1.61
Q0D840	Thioredoxin H1	9	54.92	0.49
Q6ZFU6	Thioredoxin reductase NTRB	7	41.09	1.75
Q5N9C8	Trafficking protein particle complex subunit	2	17.53	2.11
P12149	30S ribosomal protein S12, chloroplastic	2	12.90	0.54
P0C485	30S ribosomal protein S3, chloroplastic	10	32.64	0.58
Q10N98	33 kDa secretory protein, putative, expressed	6	32.45	0.10
Q0IQF7	40S ribosomal protein S16	10	47.65	0.60
Q8L4F2	40S ribosomal protein S23, putative, expressed	5	48.59	0.54
Q8LI30	4-alpha-glucanotransferase DPE1, chloroplastic/amyloplastic	6	10.94	2.17
Q2QU06	60 kDa chaperonin alpha subunit	40	57.96	0.61
Q2QNF3	60S ribosomal protein L2, putative, expressed	17	45.98	0.61
P35684	60S ribosomal protein L3	21	36.50	0.63
A0A0N7KH91	Os03g0345700 protein (Fragment)	1	6.67	0.10
A0A0P0VRK8	Os02g0818000 protein (Fragment)	6	28.70	0.23
A0A0P0VUM8	Os03g0213100 protein (Fragment)	6	10.11	2.21
Q0DSD6	Os03g0315800 protein (Fragment)	22	37.69	0.65
A0A0P0WKQ1	Os05g0323800 protein (Fragment)	9	20.46	1.51
A0A0P0WTX9	Os06g0214850 protein (Fragment)	10	38.92	1.75
A0A0P0X3W0	Os07g0237100 protein (Fragment)	5	13.53	1.84
C7JA48	Os12g0478100 protein (Fragment)	2	18.49	5.30
Q7XVP0	OSJNBa0023J03.8 protein	4	21.45	3.77
Q7X7H3	OSJNBa0076N16.12 protein	17	29.04	1.64
Q7XW32	OSJNBb0062H02.10 protein	16	56.94	0.48
Q7X6F6	OSJNBb0079B02.3 protein	18	25.23	0.42
Q0JR27	Os01g0124100 protein	5	24.32	0.12
Q0JQZ2	Os01g0130400 protein	4	4.72	0.70
Q9SDK4	Os01g0254000 protein	6	35.23	2.03
Q0JN91	Os01g0314800 protein	1	8.60	12.71
Q943W1	Os01g0501800 protein	36	66.07	1.82
Q5VP66	Os01g0644000 protein	3	22.31	0.14
Q5N754	Os01g0815800 protein	8	25.93	0.59
Q943L0	Os01g0839900 protein	6	26.32	0.41
Q943K1	Os01g0869800 protein	14	37.31	2.78
Q6YU90	Os02g0101500 protein	42	81.61	1.27
Q6EUK5	Os02g0234500 protein	17	35.43	1.96
Q6ZH84	Os02g0593700 protein	3	4.73	2.78
Q6K9C2	Os02g0610700 protein	5	17.95	1.84
Q6K1Q6	Os02g0622300 protein	11	41.79	0.72
Q6Z8I7	Os02g0752200 protein	12	19.36	0.61
Q6K3F7	Os02g0812400 protein	3	4.51	2.42
Q10N92	Os03g0278200 protein	5	9.78	2.25
A0A0N7KH54	Os03g0311300 protein	6	27.45	1.79
Q10K10	Os03g0401100 protein	1	1.73	0.59
Q94H99	Os03g0761000 protein	5	32.64	0.37
A0A0P0W5A9	Os03g0841900 protein	4	6.98	1.33
Q84M68	Os03g0856500 protein	9	24.60	0.26
B9F813	Os04g0234600 protein	38	57.91	1.77
Q7XIK5	Os04g0613600 protein	9	46.58	0.60
B9FM04	Os05g0104650 protein	13	10.74	0.64
Q0DK70	Os05g0188100 protein	4	42.70	0.33
Q75IK4	Os05g0209600 protein	8	23.82	0.34
A0A0P0WKD6	Os05g0291700 protein	41	69.29	1.80
Q0DG76	Os05g0549100 protein	18	33.45	0.47
Q0DG31	Os05g0556100 protein	18	28.41	1.54
Q0DFD6	Os05g0597100 protein	5	17.17	0.59
Q9LWT6	Os06g0114000 protein	57	67.89	0.44
Q0DEF1	Os06g0157000 protein	9	32.84	0.32
Q8GTK4	Os07g0141400 protein	25	60.63	1.67
Q84PB5	Os07g0148900 protein	6	22.56	4.02
Q6ZLQ0	Os07g0150100 protein	8	9.44	1.26
Q6ZLB8	Os07g0180900 protein	31	60.00	0.66
Q0D5S1	Os07g0539900 protein	14	28.24	0.29
Q6YPF2	Os08g0120500 protein	23	34.08	0.52
Q7EYM8	Os08g0379400 protein	24	56.41	2.51
Q6Z8N9	Os08g0512400 protein	8	23.73	0.44
A0A0P0XX30	Os10g0530500 protein	10	56.00	1.80
Q2QZH3	Os11g0687100 protein	12	16.43	2.15
A0A0P0Y5F2	Os11g0687200 protein	8	12.37	1.82
Q0IPL3	Os12g0189300 protein	15	50.00	3.37
Q2QWN3	Os12g0189400 protein	13	53.02	0.72
Q2QNS7	Os12g0555500 protein	10	70.25	2.58

*^a^* Peptides (95%) indicate the identified peptides having at least 95% confidence. *^b^* Cov (95%) indicate percentage of matching amino acids from identified peptides having at least 95% confidence.

**Table 3.** List of differentially expressed proteins after cold stress treatment for 72 h

Uniprot_ID	Putative function	Peptides (95%)*^a^*	Cov (95%)*^b^*	Fold change
Q0J709	ACT domain-containing protein DS12, chloroplastic	11	44.17	2.03
Q10P83	Acyl-CoA-binding domain-containing protein 5	1	4.39	1.94
A0A0P0Y1Y5	Adenosylhomocysteinase (Fragment)	30	55.15	1.27
Q7XYS3	Allene oxide synthase 2	12	29.08	1.72
Q9FXT4	Alpha-galactosidase	11	24.66	0.43
Q10A56	Alpha-mannosidase	20	22.71	0.67
Q5WAB3	AP-1 complex subunit gamma	8	11.03	0.72
P0C2Z6	ATP synthase subunit alpha, chloroplastic	36	51.68	1.20
P0C522	ATP synthase subunit alpha, mitochondrial	31	53.24	1.71
Q93VT8	ATP-citrate synthase beta chain protein 1	25	46.71	1.43
Q5Z974	ATP-dependent zinc metalloprotease FTSH 1, chloroplastic	34	40.38	1.32
Q655S1	ATP-dependent zinc metalloprotease FTSH 2, chloroplastic	35	47.34	1.58
Q0JR25	Bowman-Birk type bran trypsin inhibitor	8	33.86	0.39
Q2QMX9	Calcium-transporting ATPase 10, plasma membrane-type	7	6.76	1.43
B9EXM2	Carbamoyl-phosphate synthase large chain, chloroplastic	29	26.37	0.65
Q75HY2	Carboxypeptidase	11	31.45	1.56
Q10RP0	Cell division cycle protein 48, putative, expressed	39	53.40	1.79
Q84T92	Chalcone—flavonone isomerase	11	53.65	2.51
Q6H795	Chaperone protein ClpD1, chloroplastic	11	14.29	3.70
Q6H748	Chlorophyll a-b binding protein, chloroplastic	16	31.97	2.00
Q7XV11	Chlorophyll a-b binding protein, chloroplastic	15	48.41	2.13
Q53N83	Chlorophyll a-b binding protein, chloroplastic	22	51.59	1.38
Q6ZF30	Chlorophyll a-b binding protein, chloroplastic	10	51.13	3.08
Q10HD0	Chlorophyll a-b binding protein, chloroplastic	16	45.25	2.73
Q5W6F1	Cinnamate-4-hydroxylase	8	15.00	2.05
Q6ZGV8	Clustered mitochondria protein homolog	8	7.36	1.53
Q6YUR8	Cold shock domain protein 1	11	68.88	0.48
Q7XCS3	Cys/Met metabolism PLP-dependent enzyme family protein, expressed	5	11.42	3.16
Q7XKC8	Dihydroorotate dehydrogenase (quinone), mitochondrial	5	13.22	0.25
Q306J3	Dirigent protein	16	58.50	1.82
Q69JX7	Drought-induced S-like ribonuclease	3	13.10	0.49
Q2QN11	Eukaryotic aspartyl protease family protein, expressed	10	25.34	0.43
Q7G649	Expressed protein	15	61.82	0.52
Q2R678	Expressed protein	11	27.67	1.54
Q10T66	Expressed protein	9	49.75	0.51
Q2R8Z8	Expressed protein	1	13.86	14.32
P41344	Ferredoxin—NADP reductase, leaf isozyme 1, chloroplastic	30	56.08	1.77
Q6ZFJ3	Ferredoxin—NADP reductase, leaf isozyme 2, chloroplastic	29	54.37	2.23
Q6ZD89	Flavone 3ʹ-O-methyltransferase 1	20	60.60	1.96
Q69V57	Fructose-bisphosphate aldolase	31	75.14	1.42
Q40677	Fructose-bisphosphate aldolase, chloroplastic	47	57.73	1.47
Q5VQG4	Galactinol—sucrose galactosyltransferase OS = *Oryza sativa* subsp. *japonica* OX = 39947 GN = RFS PE = 1 SV = 1	6	8.94	2.49
Q6Z2T6	Geranylgeranyl diphosphate reductase, chloroplastic	26	47.08	0.64
P15280	Glucose-1-phosphate adenylyltransferase small subunit 2, chloroplastic/amyloplastic/cytosolic	22	41.05	1.33
P14656	Glutamine synthetase cytosolic isozyme 1-1	9	33.99	1.74
Q945W2	Glutathione S-transferase GSTU6, putative, expressed	6	28.81	2.15
Q8H8D6	Glutathione S-transferase, N-terminal domain containing protein, expressed	16	46.39	0.62
Q0J8A4	Glyceraldehyde-3-phosphate dehydrogenase 1, cytosolic	28	62.02	2.81
A3C6G9	Glycine cleavage system H protein, mitochondrial	8	67.68	0.41
Q10CU9	Glycosyl hydrolase family 3 N terminal domain containing protein, expressed	17	28.32	0.60
Q7XU02	Glycosyltransferase	3	7.34	2.65
Q5VME5	Glycosyltransferase	11	31.19	1.82
Q7XUC9	Histone H4	12	58.25	2.96
Q851P9	Histone-like protein	6	19.11	3.73
O64437	Inositol-3-phosphate synthase 1	4	8.43	2.40
Q84TA3	Leucine aminopeptidase	15	25.20	0.65
Q6K669	Leucine aminopeptidase 2, chloroplastic	27	39.63	1.29
P38419	Lipoxygenase 7, chloroplastic	16	18.94	1.54
Q7XZW5	Malate dehydrogenase	34	85.31	1.64
Q2QM23	Methyl-CpG binding domain containing protein, expressed	13	56.44	0.52
Q7XUK3	NADPH oxidoreductase	10	36.23	3.70
Q0D5P8	Oxygen-evolving enhancer protein 3, chloroplastic	22	58.06	1.25
Q5JMS4	Peroxidase	18	50.40	1.79
Q6AVZ8	Peroxidase	16	49.12	0.44
Q9FYP0	Peroxidase	10	31.99	0.48
Q0JM38	Peroxidase	8	25.59	0.27
Q5Z7J2	Peroxidase	13	30.86	0.47
Q0DCP0	Peroxidase (Fragment)	14	39.53	0.50
A0A0P0XR31	Peroxidase (Fragment)	12	32.14	0.57
Q6YT73	Peroxisomal (S)-2-hydroxy-acid oxidase GLO5	38	65.85	2.01
P14717	Phenylalanine ammonia-lyase	44	55.35	2.70
Q6K6Q1	Phenylalanine ammonia-lyase	23	30.08	3.98
Q0DZE0	Phenylalanine ammonia-lyase	22	27.91	5.86
Q75W16	Phospho-2-dehydro-3-deoxyheptonate aldolase 2, chloroplastic	14	25.60	2.49
A0A0P0WP33	Phosphoglycerate kinase	41	68.18	1.92
P0C355	Photosystem I P700 chlorophyll a apoprotein A1	19	16.80	1.56
P0C358	Photosystem I P700 chlorophyll a apoprotein A2	20	20.84	2.56
P0C364	Photosystem II CP47 reaction center protein	24	31.89	2.75
Q8GT95	Polygalacturonase inhibitor 1	8	32.83	0.43
Q5ZCK5	Probable calcium-binding protein CML16	5	31.49	2.05
Q75L11	Probable histone H2A.6	6	30.13	4.41
Q6K439	Probable plastid-lipid-associated protein 2, chloroplastic	17	42.01	1.79
Q0D5W6	Protein translation factor SUI1 homolog	6	43.48	2.68
Q6ZHE5	Putative D-cysteine desulfhydrase 1, mitochondrial	13	34.98	1.34
Q7XKB5	Pyruvate kinase	18	32.09	1.22
P31924	Sucrose synthase 1	63	54.90	1.33
Q93X08	UTP—glucose-1-phosphate uridylyltransferase	35	60.55	1.39
Q06967	14-3-3-like protein GF14-F	28	74.62	1.43
Q6ER94	2-Cys peroxiredoxin BAS1, chloroplastic	17	49.81	1.45
P0C488	30S ribosomal protein S4, chloroplastic	11	42.29	2.03
Q8LI30	4-alpha-glucanotransferase DPE1, chloroplastic/amyloplastic	6	10.94	2.09
Q2QLY5	5-methyltetrahydropteroyltriglutamate—homocysteine methyltransferase 1	40	43.99	2.03
P49210	60S ribosomal protein L9	11	55.26	1.54
Q2R480	6-phosphogluconate dehydrogenase, decarboxylating 2, chloroplastic	17	37.20	1.43
A0A0N7KH91	Os03g0345700 protein (Fragment)	1	6.67	0.12
A0A0P0VRK8	Os02g0818000 protein (Fragment)	6	28.70	0.29
A0A0P0VTG7	Os03g0165300 protein (Fragment)	4	8.65	0.18
A0A0P0VUM8	Os03g0213100 protein (Fragment)	6	10.11	2.38
Q0DEU8	Os06g0133800 protein (Fragment)	52	64.45	1.50
A0A0P0Y445	Os11g0602500 protein (Fragment)	13	37.32	1.71
C7JA48	Os12g0478100 protein (Fragment)	2	18.49	11.91
Q7XPV4	OSJNBa0088H09.2 protein	15	38.22	0.68
Q7XVF8	OSJNBb0118P14.7 protein	5	15.38	2.00
Q94EG1	Os01g0178000 protein	7	24.37	0.69
Q5VR43	Os01g0180300 protein	51	55.48	0.61
Q9SDK4	Os01g0254000 protein	6	35.23	1.57
Q9FP25	Os01g0303000 protein	14	62.90	0.44
Q0JN91	Os01g0314800 protein	1	8.60	8.32
Q6YU90	Os02g0101500 protein	42	81.61	1.58
Q6YUV4	Os02g0189000 protein	6	32.88	0.51
Q6EUK5	Os02g0234500 protein	17	35.43	1.67
Q6ZH84	Os02g0593700 protein	3	4.73	4.83
Q6Z8I7	Os02g0752200 protein	12	19.36	0.62
Q0DWG3	Os02g0816300 protein	4	3.93	0.40
Q10RI4	Os03g0158500 protein	5	8.77	2.17
Q10N92	Os03g0278200 protein	5	9.78	2.25
Q9AUQ4	Os03g0712700 protein	32	51.03	1.42
Q8S7H8	Os03g0778100 protein	11	37.29	2.29
B9F813	Os04g0234600 protein	38	57.91	1.89
Q0JAF4	Os04g0602100 protein	19	43.63	1.77
Q75IR7	Os05g0163000 protein	15	26.73	1.46
Q0DK70	Os05g0188100 protein	4	42.70	0.42
Q75IK4	Os05g0209600 protein	8	23.82	0.57
Q6F322	Os05g0490700 protein	9	13.84	1.33
Q0DG76	Os05g0549100 protein	18	33.45	0.51
Q9SNN5	Os06g0130800 protein	8	30.83	0.69
Q7XXQ8	Os06g0232000 protein	6	16.80	0.36
Q69WH2	Os06g0332800 protein	6	44.27	0.43
Q93W07	Os06g0568200 protein	23	50.82	1.24
Q67W57	Os06g0646500 protein	17	56.00	2.03
Q5YM05	Os06g0720400 protein	10	22.75	1.58
Q8GTK4	Os07g0141400 protein	25	60.63	1.57
Q84PB5	Os07g0148900 protein	6	22.56	4.66
A0A0P0X7J0	Os07g0539400 protein	6	11.32	0.40
Q0D5S1	Os07g0539900 protein	14	28.24	0.63
Q0D3Z0	Os07g0658300 protein	1	1.03	0.72
Q6ZFI6	Os08g0502700 protein	29	58.21	1.82
Q650W6	Os09g0565200 protein	13	36.65	0.54
Q2QZH3	Os11g0687100 protein	12	16.43	1.67
A0A0P0Y5F2	Os11g0687200 protein	8	12.37	1.72
Q0IPL3	Os12g0189300 protein	15	50.00	2.51
Q2QSR7	Os12g0420200 protein	32	55.05	1.33

*^a^* Peptides (95%) indicate the identified peptides having at least 95% confidence. *^b^* Cov (95%) indicate percentage of matching amino acids from identified peptides having at least 95% confidence.
